# Thalamic Reorganization in Chronic Patients With Intracerebral Hemorrhage: A Retrospective Cross-Sectional Study

**DOI:** 10.1097/MD.0000000000001391

**Published:** 2015-08-28

**Authors:** Sung Ho Jang, Chul Hoon Chang, Seong Ho Kim, Young Jin Jung, Ji Heon Hong

**Affiliations:** From the Department of Physical Medicine and Rehabilitation (SHJ); Departments of Neurosurgery, College of Medicine, Yeungnam University (CHC, SHK, YJJ); and Department of Physical Therapy, Sun Moon University, Asan-si, Chungnam, Republic of Korea (JHH).

## Abstract

The aim of this study was to investigate changes of synaptic area of the spinothalamic tract and its thalamocortical pathway (STT) in the thalamus in chronic patients with putaminal hemorrhage.

Twenty four patients with a lesion in the ventral posterior lateral nucleus (VPL) of the thalamus following putaminal hemorrhage were recruited for this study. The subscale for tactile sensation of the Nottingham Sensory Assessment (NSA) was used for the determination of somatosensory function. Diffusion tensor tractography of the STT was reconstructed using the Functional Magnetic Resonance Imaging of the Brain Software Library. We classified patients according to 2 groups: the VPL group, patients whose STTs were synapsed in the VPL; and the non-VPL group, patients whose STTs were synapsed in other thalamic areas, except for the VPL.

Thirteen patients belonged to the VPL group, and 8 patients belonged to the non-VPL group. Three patients were excluded from grouping due to interrupted integrity of the STTs. The tactile sensation score of the NSA in the non-VPL group (10.50 ± 0.93) was significantly decreased compared with that of the VPL group (19.45 ± 1.33) (*P* < 0.05).

We found that 2 types of patient had recovered via the VPL area or other areas of the STT. It appears that patients who showed shifting of the thalamic synaptic area of the STT might have recovered by the process of thalamic reorganization following thalamic injury. In addition, thalamic reorganization appears to be related to poorer somatosensory outcome.

## INTRODUCTION

Somatosensory function has important implications in terms of high incidence of somatosensory dysfunction, association with functional impairment, and safety in stroke patients.^1–3^ Therefore, elucidation of the mechanism for recovery of somatosensory dysfunction would be important for stroke patients in the development of guidelines for successful rehabilitation. However, relatively little is known about the mechanisms for recovery of somatosensory dysfunction in stroke patients compared with other functions, such as motor function.^1^ According to previous studies, the mechanisms for the recovery of somatosensory function can be summarized as follows: recovery of an injured somatosensory neural tract, perilesional reorganization at the cortex or subcortical level, contribution of the unaffected somatosensory cortex, and contribution of the secondary somatosensory cortex. However, the majority of these studies reported on recovery of injured somatosensory tracts or cortical reorganization, and relatively little is known about subcortical reorganization.^1,4–8^

It is well-known that 2 main somatosensory tracts are exist in the human brain: the medial lemniscus and its related thalamocortical pathway is involved in proprioception, and the spinothalamic tract and its related thalamocortical pathway (STT) is responsible for pain, touch, and body temperature.^9,10^ Recently, diffusion tensor tractography (DTT), which is derived from DTI, allows reconstruction and estimation of the somatosensory tracts in the live human brain.^11,12^ In particular, DTT using a probabilistic tractography algorithm has been known to have advantages in investigation of the anatomical location of a neural tract.^13,14^ Some studies have reported on the clinical usefulness of demonstration of an injured somatosensory neural traction stroke patients.^15,16^ However, no study on subcortical reorganization has been reported.

In the current study, using DTT, we attempted to investigate changes of the synaptic area of the STT in the thalamus in chronic patients with putaminal hemorrhage (PH).

## METHODS

### Subjects

Twenty four patients with intracerebral hemorrhage (ICH) (13 males, 11 females; mean age 49.73 ± 8.23 years) were recruited according to the following criteria: first ever stroke, more than 6 months after ICH onset, a hematoma located primarily in the lentiform nucleus of the basal ganglia and a lesion with involvement in the ventral posterior lateral nucleus (VPL) of the thalamus, confirmed by a neuroradiologist, and with no evidence of other serious associative problems (e.g., aphasia, attention deficits, Mini-Mental State Examination <25, and visual neglect). The subscale for tactile sensation (full mark: 20) of the Nottingham Sensory Assessment (NSA) was used for the determination of somatosensory function at the time of DTI scanning.^17^ The reliability and validity of the NSA scale are well-established.^17^ This study was conducted retrospectively, and the study protocol was approved by the Institutional Review Board of our hospital.

### Data Acquisition and Fiber Tracking

A 1.5-T Philips Gyroscan Intera system (Hoffman-LaRoche, Ltd., Best, The Netherlands) was used for DTI scanning at an average of 13.96 months (±12.82) after ICH onset. Sixty seven contiguous slices parallel to the anterior commissure–posterior commissure line were acquired for each of the 32 noncollinear diffusion sensitizing gradients. DTI imaging parameters were matrix-128 × 128, FOV-221 × 221 mm^2^, TR/TE/NEX-10726/76/1, synergy-L Sensitivity Encoding factor-2; echoplanar imaging factor-67 and b-1000 s/mm^2^, slice thickness-2.3 mm, and voxel size-2.3 × 2.3 × 2.3 mm^3^.

**FIGURE 1:**
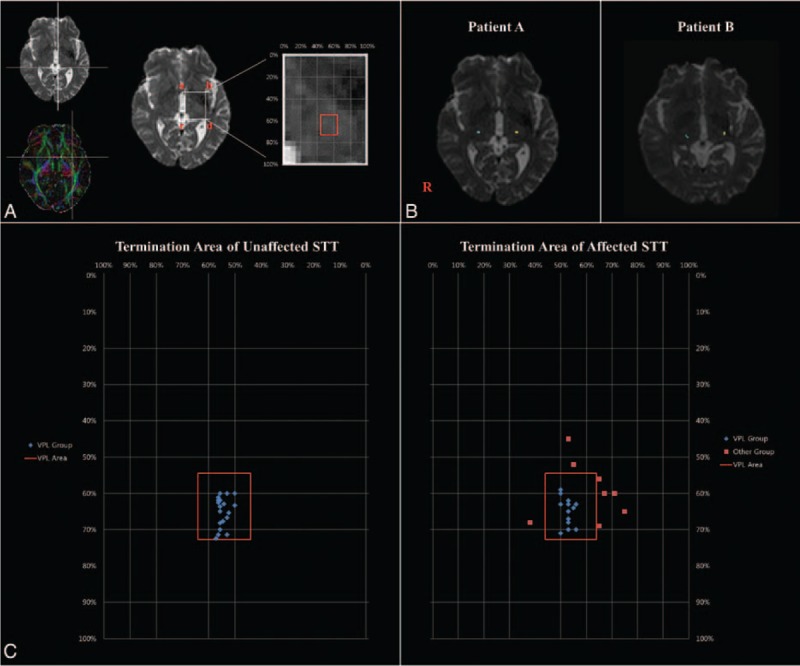
(A) Landmarks for measurement of the main termination area. The line, ab, passing the anterior commissure, was defined as the anterior boundary, and the line, cd, which passed the most posterior point of the thalamus, as the posterior boundary. The ac line was defined as the medial boundary, passing the most medial point of the thalamus, and the bd line passing the most lateral point of the thalamus, as the lateral boundary. (B) Patient A: The STT passed through the VPL. Patient B: The STT ascended to the non-VPL of the thalamus in the affected hemisphere. (C) Map of the STT in the thalamus. The blue spot indicates the VPL group, which passed through the VPL (red quadrangle). The red spot indicates the non-VPL group for individual subjects. STT = spinothalamic tract and its thalamocortical pathway, VPL = ventral posterior lateral nucleus.

The Oxford Centre for Functional Magnetic Resonance Imaging of the Brain Software Library (FSL; www.fmrib.ox.ac.uk/fsl) was used for the analysis of DTI data. Affine multiscale 2-dimensional registration was used to remove eddy current-induced image distortions and motion artifacts.^18^ A probabilistic tractography method based on a multifiber model was used for fiber tracking (5000 streamline samples, 0.5-mm step lengths, and curvature threshold 0.2).^13,19^ The spinothalamic tract of the posterolateral medulla (posterior to the inferior olivary nucleus, anterior to the inferior cerebellar peduncle, and lateral to the medial lemniscus) was selected for the seed mask.^20–22^ A waypoint mask was placed in one-third of the lateral portion and one-third of the anterior portion on the blue area of the pons posterior to the transpontine fiber was selected for the waypoint mask (Figure 1).^12,20,23–26^

### Measurements of Location of the STT in the Thalamus

Location of the STT (x, y) in the thalamus was selected at the highest probability point for each subject in the bicommissural level. The level was the first axial image that can be seen on both the anterior and posterior commissures. The highest point is the largest number of fibers passing through a given voxel in the STT at the thalamus level. As shown in Figure 1, the ab line, passing the anterior commissure, was defined as the anterior boundary, and the line, cd, which passed the most posterior point of the thalamus, as the posterior boundary (Figure 1). The ac line was defined as the medial boundary, passing the most medial point of the thalamus, and the bd line passing the most lateral point of the thalamus, as the lateral boundary. Each location of the point was calculated as an individual pixel unit. We measured the location of the STT at the thalamus using the following equation: 
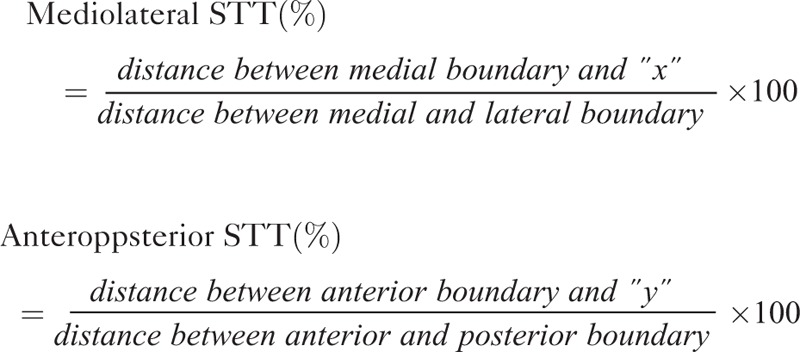


Patients were classified into 2 groups according to synaptic area of the STT in the thalamus. The STT of the VPL group passed the VPL of the thalamus, which was defined from 54.5% to 72.7% in the anteroposterior direction and from 40.0% to 64.0% in the mediolateral direction.^27^ Patients whose STTs were synapsed in other thalamic areas, except for the VPL, were assigned to the non-VPL group.

### Statistical Analysis

Statistical analysis was performed using SPSS 12.0 for Windows (SPSS Inc., Chicago, IL). Test selection was based on evaluating the variables for normal distribution using the Kolmogorov–Smirnov test. If the variables had a normal distribution, independent *t*-test was used. If the variable did not have a normal distribution, the analysis was done using the Mann–Whitney *U* test. To comparison of gender was evaluated by Chi-square test. Statistical significance was chosen as *P* *<* 0.05.

## RESULTS

The reconstructed STT started from the posterolateral medulla, which was selected as a seed mask, and ascended to the thalamus, through the pontine tegmentum. Subsequently, the thalamocortical fibers originating from the VPL of the thalamus ascended through the posterior limb of the internal capsule and the posterior portion of the corona radiata, terminating in the postcentral gyrus. Our results showed that there was no group difference in the distribution of gender and age. By contrast, the NSA (tactile sensation) and duration after onset were not normally distributed. No significant difference observed in the duration after onset between the 2 groups (*P* > 0.05). Thirteen patients (6 males, 7 females; mean age 50.00 ± 8.30 years) belonged to the VPL group, and 8 patients (2 males, 6 females; mean age 53.63 ± 6.52 years) belonged to the non-VPL group (Table 1). Three patients were excluded from grouping due to interrupted integrity of the STTs. The relative average location of the highest probability point of the STT for the affected hemisphere was 65% (VPL group) and 59% (non-VPL group) in the mediolateral direction, and 53% (VPL group) and 61% (non-VPL group) in the anteroposterior direction. The synaptic area of the STT for affected hemisphere showed significant difference between the groups (*P* < 0.05). In the non-VPL group, the STT passed through the anterior to the VPL of the thalamus in 2 patients and lateral to the VPL in 5 patients (Figure 1). In contrast, there was no significant difference in the synaptic area of the STT between the VPL group and the non-VPL group for the unaffected hemisphere (*P* > 0.05). Average score for the tactile sensation of the NSA (full score: 20) was 19.45 ± 1.33 in the VPL group and 10.50 ± 0.93 in the non-VPL group, respectively, the tactile sensation score for the NSA in the non-VPL group was significantly lower compared with that of the VPL group (*P* < 0.05).

**TABLE 1:**
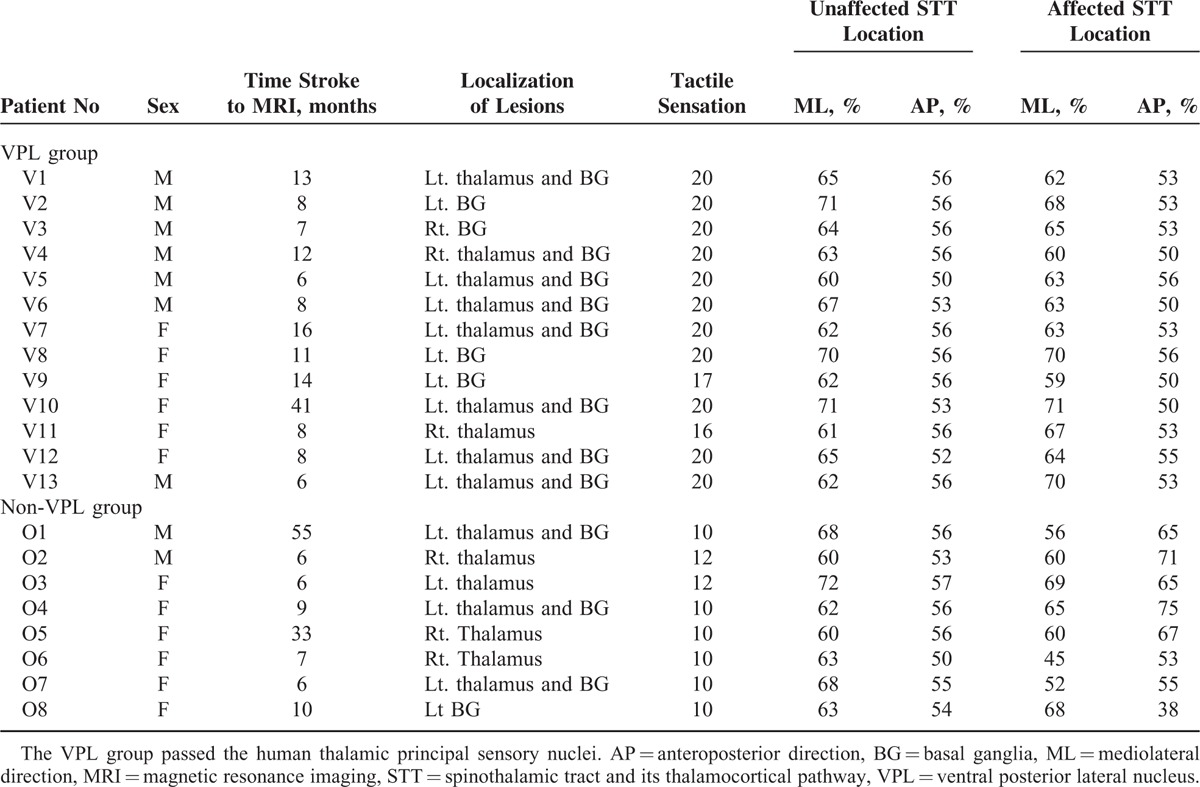
Clinical Characteristics and Location of the Main Termination Area for the STT

## DISCUSSION

In the current study, we attempted to find changes in the location of the thalamic synapse of the STT in chronic patients with involvement of the thalamic VPL due to PH. The thalamic synaptic area was selected according to the highest probability point using a probabilistic tractography method based on a multifiber model. The value of probability could represent the strength of connection or the number of fibers.^14,19,28^ We divided the patients into 2 groups according to the thalamic synaptic area of the STT, the VPL group and the non-VPL group. As a result, we believe that in patients in the VPL group, the injured STT was recovered by the original synaptic area (VPL) of the thalamus after thalamic injury by PH. By contrast, in non-VPL patients, it appeared that the injured STT was recovered by shifting the synapse into another thalamic area, except for the VPL. Therefore, the shifting of the synapse to another thalamic area appears to suggest thalamic reorganization following thalamic injury due to PH.

On the other hand, significantly lower somatosensory function was observed in patients in the non-VPL group compared with the VPL group, although patients in the VPL group showed a subnormal score of NSA (19.45, full score: 20), nearly half of the tactile sensation score of the NSA (10.50) was observed in the non-VPL group. Consequently, it appears that thalamic reorganization of the STT accompanied poorer somatosensory function. We believe that these results coincide with those of previous studies showing that patients who recovered by reorganization or other pathways, except for normal neural tracts, showed poorer function than those who recovered by a normally existing neural pathway.^1,4,6,8,29^

The thalamus, a synaptic station of the ascending somatosensory pathway, transfers somatosensory input to the cerebral cortex. Therefore, independent involvement of the thalamus in plasticity of the somatosensory system has been suggested.^30–32^ Several studies have reported on mechanisms of somatosensory recovery in stroke patients with thalamic lesions, using functional magnetic resonance imaging (fMRI) or microelectrode stimulation study.^4–7^ Regarding fMRI, in 2002, Staines et al^6^ demonstrated the mechanism of somatosensory recovery in 4 patients with thalamic lesions due to stroke (2 patients: infarct and 2 patients: hemorrhage). They performed fMRI during somatosensory stimulation from an early to a chronic stage of stroke and found an association of somatosensory recovery with enhancement of activation of the affected primary somatosensory cortex. Subsequently, Lee et al^4^ (2011) reported fMRI findings of 11 chronic patients with thalamic hemorrhage. They reported a positive association of proprioception with relative activity in the ipsilateral versus the contralateral primary sensorimotor cortex of the affected hand, and concluded that recovery of the proprioceptive function of the affected hand occurred through normally existing medial lemniscus and its related thalamocortical pathway. However, the above-mentioned fMRI studies could not demonstrate changes at the subcortical level. Regarding changes at the subcortical level, a few studies have reported on the use of microelectrode stimulation study.^5,7^ In 1985, Ohye et al^7^ investigated change of a neuronal receptive field for somatosensory stimuli. They recruited 7 patients with tremor after stroke (location of lesion: 2 patients – thalamus, the remaining patients – other areas). A total of 21 receptive fields in the thalamus reacted to repeated application of tactile or kinesthetic stimulation in the body. The receptive fields were distributed in and around the human thalamic principal sensory nucleus. The reorganized receptive fields for tactile stimulation were located latero-posteriorly to the principal sensory nucleus. By contrast, the reorganized receptive fields for kinesthetic neurons shifted to the anterior principal sensory nucleus. In 2001, Ohara and Lenz^5^ reported on a microelectrode stimulation study of a patient with a thalamic infarct in whom reorganization of the somatosensory nucleus of the thalamus into other thalamic nuclei located anterior to the infarct was observed. As a result, to the best of our knowledge, this is the first study to demonstrate the reorganization of the STT in stroke patients with thalamic lesion, using DTI.

In conclusion, we investigated changes of the thalamic synaptic area of the STT after a VPL lesion due to PH. We identified that the types of patients who had recovered via the VPL area or other areas of the STT. It appears that patients who showed shifting of the thalamic synaptic area of the STT might have recovered by the process of thalamic reorganization of the synaptic area of the STT following thalamic injury. In addition, the touch sensation of patients who showed shifting of the synaptic area was decreased compared with that of patients who showed no shifting. As a result, thalamic reorganization appears to be related to poorer somatosensory outcome. We believe that limitations of this study should be considered in interpretation of the results. First, this study investigated the changes of synaptic area of the STT based on in vivo DTI. The difference of synaptic location in patients who have recovered by the process of reorganization with the VPL was relatively larger, as compared with voxel size of DTI. However, the structural morphology and tissue contrasts might be different between in vivo and ex vivo. Second, the location of the STT in the thalamus was selected as the highest probability point using the probabilistic tractography. The highest point is the largest number of fiber passing through a given voxel in the STT. However, probabilistic tractography may include false-positive results; on the contrary, DTI may underestimate fiber tracts because regions of fiber complexity and crossing can obscure the underlying fiber architecture.^19,33^ Finally, this retrospective study included a relatively small number of patients and did not include long-term follow-up. Therefore, further long-term follow-up prospective studies including a larger number of patients to elucidate the factors which are relevant with prognosis should be encouraged.
